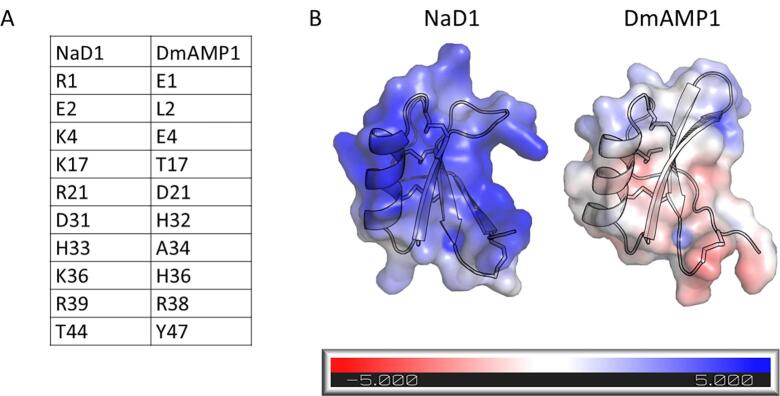# The interaction with fungal cell wall polysaccharides determines the salt tolerance of antifungal plant defensins

**DOI:** 10.1016/j.tcsw.2019.100026

**Published:** 2019-05-22

**Authors:** Mark R. Bleackley, Charlotte S. Dawson, Jennifer A.E. Payne, Peta J. Harvey, K. Johan Rosengren, Pedro Quimbar, Donovan Garcia-Ceron, Rohan Lowe, Vincent Bulone, Nicole L. van der Weerden, David J. Craik, Marilyn A. Anderson

**Affiliations:** a Department of Biochemistry and Genetics, La Trobe Institute for Molecular Science, Kingsbury Drive, La Trobe University, Bundoora, Victoria 3086, Australia; b Australian Research Council Centre of Excellence in Plant Cell Walls, School of Agriculture, Food and Wine, University of Adelaide, Waite Campus, Glen Osmond, South Australia 5064, Australia; c Division of Chemistry and Structural Biology, Institute for Molecular Bioscience, The University of Queensland, Brisbane, Queensland 4072, Australia; d School of Biomedical Sciences, Faculty of Medicine, The University of Queensland, Brisbane, Queensland 4072, Australia; e EMBL Australia, Monash University, Clayton, Victoria 3800, Australia; f The Monash Biomedicine Discovery Institute, Department of Biochemistry and Molecular Biology and ARC Centre of Excellence in Advanced Molecular Imaging, Monash University, Clayton, Victoria 3800, Australia

**Keywords:** Fungi, β-Glucan, Plant defensin, Salt

## Abstract

The fungal cell wall is the first point of contact between fungal pathogens and host organisms. It serves as a protective barrier against biotic and abiotic stresses and as a signal to the host that a fungal pathogen is present. The fungal cell wall is made predominantly of carbohydrates and glycoproteins, many of which serve as binding receptors for host defence molecules or activate host immune responses through interactions with membrane-bound receptors. Plant defensins are a large family of cationic antifungal peptides that protect plants against fungal disease. Binding of the plant defensin NaD1 to the fungal cell wall has been described but the specific component of the cell wall with which this interaction occurred was unknown. The effect of binding was also unclear, that is whether the plant defensin used fungal cell wall components as a recognition motif for the plant to identify potential pathogens or if the cell wall acted to protect the fungus against the defensin. Here we describe the interaction between the fungal cell wall polysaccharides chitin and β-glucan with NaD1 and other plant defensins. We discovered that the β-glucan layer protects the fungus against plant defensins and the loss of activity experienced by many cationic antifungal peptides at elevated salt concentrations is due to sequestration by fungal cell wall polysaccharides. This has limited the development of cationic antifungal peptides for the treatment of systemic fungal diseases in humans as the level of salt in serum is enough to inactivate most cationic peptides.

## 1 Introduction

Fungal pathogens are a serious threat to humanity because of their impact on food production and human and animal health. They are the largest contributors to the 20% yield loss and 10% post-harvest loss caused by microbial pathogens in major food crops (Bebber and Gurr, 2015, Fisher et al., 2012). In humans, fungal infections range from superficial skin and mucosal infections to serious infections of the blood stream and internal organs (Enoch et al., 2006, Gow and Netea, 2016). Antifungals used in both agriculture and medicine are directed to specific fungal targets such as the sterol ergosterol or biosynthesis of polysaccharides in the fungal cell wall (Kathiravan et al., 2012). However, emerging resistance (Anderson, 2005, Kanafani and Perfect, 2008) and host toxicity (Kathiravan et al., 2012) has created the need for novel antifungals to combat fungal disease. Naturally occurring antifungal peptides (AFPs) are a family of molecules with novel mechanisms of action that have the potential to meet this need (van der Weerden et al., 2013).

Plant defensins are one of the largest families of AFPs. These small, cationic proteins are a major component of the plant innate immune system (Parisi et al., 2018). They are characterized by a common three-dimensional structure consisting of a triple stranded β-sheet linked to an α-helix by three stabilizing disulphide bonds. A fourth disulphide bond links the N- and C-terminal regions of the defensin, rendering it pseudocyclic and contributing to the stability of the fold (van der Weerden and Anderson, 2013). Despite their conserved structure, defensins are hypervariable in sequence with only the cysteine residues and a glycine residue being completely conserved across the family (Shafee et al., 2016). This sequence diversity underlies the diverse functions, including an array of antifungal mechanisms, that have been described for plant defensins (van der Weerden and Anderson, 2013).

The cell wall is the first point of contact between an antifungal molecule and a fungal cell. Fungal cell walls are composed predominantly of a matrix of 1,3- and 1,6-β-glucans and chitin, with embedded glycoproteins and other molecules (Gow et al., 2017). The mechanical strength of the fungal cell wall makes it a key structure in protection against a range of biotic and abiotic threats. More than just an inert shell, the fungal cell wall and its dynamics are key to survival during stress (Latgé, 2007). Conversely, the uniqueness of the molecules that comprise the fungal cell wall make them key recognition motifs for both plant and animal immune systems (Gow et al., 2017).

AFPs interact with the fungal cell wall in several ways. Some plant AFPs actively break down the cell wall such as tomato osmotin AP24 and thaumatin proteins from barley which have β-glucanase activity (Osmond et al., 2001). Others interfere with the biosynthesis of the cell wall, such as hevein from the rubber tree *Hevea brasiliensis* which inhibits chitin synthase (Van Parijs et al., 1991). There are many antifungal proteins which interact with cell wall components but the role of this interaction in the inhibition of fungal growth is often not understood. These interactions occur with cell wall proteins, lipids, and carbohydrates (Fujimura et al., 2005, Koo et al., 2004, Thevissen et al., 2004).

Among the best characterized interactions between defence proteins and carbohydrates are those between proteins containing a chitin-binding domain and chitin (Raikhel et al., 1993). Chitin binding domains contain a series of conserved glycine and cystine residues in three or four disulphide bonds (Raikhel et al., 1993). Often, as in wheat germ agglutinin, proteins have multiple chitin binding domains and form dimers that allow the protein to bind to more than one chitin chain, agglutinate the carbohydrate and inhibit fungal growth (Mirelman et al., 1975). Smaller proteins such as antimicrobial peptides from *Amaranthus caudatus* (Broekaert et al., 1992) or hevein (Van Parijs et al., 1991) have a single chitin-binding domain but still bind chitin. These proteins do not agglutinate the carbohydrate, but instead use chitin segments as recognition motifs to target activities against fungal pathogens. The interaction between a thaumatin-like protein from barley with 1,3-β-glucan has been characterized in some detail. The interaction is pH specific and molecular modelling has been used to predict the carbohydrate binding site on the protein (Osmond et al., 2001).

The plant defensin NaD1 from the ornamental tobacco *Nicotiana alata* (Lay et al., 2003a) has potent antifungal activity against a number of plant (van der Weerden et al., 2008) and human (Hayes et al., 2013) pathogens. NaD1 binds to the fungal cell surface and then enters the cytoplasm of fungal cells (van der Weerden et al., 2008) by endocytosis (Hayes et al., 2018) before killing the fungal cell via the production of reactive oxygen species and permeabilization of the fungal cell membrane (Hayes et al., 2013, van der Weerden et al., 2010). Prior to entering the cytoplasm and exerting antifungal activity NaD1 must cross the fungal cell wall. Confocal microscopy using fluorescently labelled NaD1, immunofluorescence detection using a fluorescently labelled anti-NaD1 antibody and Western blotting with anti-NaD1 antibody on extracts from cell walls isolated from NaD1 treated fungi demonstrated that the defensin accumulates in the fungal cell wall (Hayes et al., 2018, Hayes et al., 2013, van der Weerden et al., 2008). Enzymatic removal of the β-glucan or protein component of the cell wall from *Fusarium oxysporum* hyphae protected cells from the antifungal activity of NaD1 (van der Weerden et al., 2010) leading to the hypothesis that an interaction between NaD1 and a fungal cell wall component was required for antifungal activity. However, NaD1 and the other defensins assessed here do not have any sequence similarity to chitin-binding domains from other proteins. Here, we describe the interaction between NaD1 and other plant defensins with the major fungal cell wall polysaccharides β-glucan and chitin. Different defensins displayed different polysaccharide binding patterns, explaining in part the variation in activity against different fungal species with different cell wall compositions. Decreasing the levels of the major cell wall polysaccharides by both chemical and genetic means revealed that yeast β-glucan had a protective effect against the antifungal activity of defensins while chitin levels had only a minimal effect. This supports the observation that the activity of plant defensins is enhanced when used in combination with β-glucan synthesis inhibitors. Additionally, enzymatic removal of the fungal cell wall restored the activity of NaD1 in the presence of salt. This indicates that NaD1 is inactivated by interaction with cell wall components at physiological salt concentrations and explains why some defensins are inactivated by salt and are less active in biological fluids. Defensins that do not interact with cell wall polysaccharides are thus more likely to retain antifungal activity in high salt environments.

## 2 Methods

### 2.1 Source of protein

NaD1 and NaD2 were isolated from the flowers of *N. alata* as described in (Lay et al., 2003a). ^15^N labelled NaD1 was produced in *Pichia pastoris* as described in (Chen et al., 2006) and purified by cation-exchange chromatography and RP-HPLC as described in (Hayes et al., 2014). HXP4 (Bleackley et al., 2017), DmAMP1 (Osborn et al., 1995) and TsD10 were expressed in *Pichia pastoris* and purified by cation-exchange chromatography and RP-HPLC as described in (Hayes et al., 2013, Lay et al., 2012).

### 2.2 Strains

*Saccharomyces cerevisiae* deletion strains were retrieved from the haploid non-essential deletion collection (ThermoScientific) and were isogenic with the wild type BY4741 (MATa *his3*Δ*1 leu2*Δ*0 met15*Δ*0 ura3*Δ*0*). *Candida albicans* experiments were performed with strain ATCC90028.

### 2.3 Polysaccharide binding

Defensin binding to polysaccharides was examined using the protocol described in (Osmond et al., 2001) with some modifications. Briefly, the insoluble polysaccharides, yeast β-glucan (Megazyme), chitin from shrimp shells (Sigma), and microcrystalline cellulose (Sigma) were washed twice with water and once with phosphate buffered saline (PBS) and resuspended in PBS at 100 mg/mL. The appropriate volume of polysaccharide suspension was transferred to a 1.5 mL microfuge tube. PBS was added to increase the volume to 47.5 µL followed by defensin (2.5 µL of 0.8 mg/mL in H_2_O). This mixture was incubated on a rotating wheel at room temperature for 30 min before insoluble polysaccharide was pelleted at 17 000 g. Supernatant (25 µL) was transferred to a clean 1.5 mL microfuge tube, mixed with 5 µL of a 1:9 mix of Bond Breaker reducing agent (ThermoFisher):NuPage LDS Loading Buffer (Invitrogen) and heated to 95 ˚C for 20 min. Samples (20 µL) were loaded onto a 10% NOVEX Bolt BisTris gel (Life Technologies). Proteins were visualized by staining with RAPIDstain (Calbiochem) and imaged on a ChemiDoc (Bio-Rad). Densitometry was performed on resulting bands using the quantitation tool in ImageLab (V4.0 Bio-Rad) and the abundance of defensin remaining in the supernatant was calculated relative to the no polysaccharide control. Relative binding to the pellet was inferred as the amount of defensin that had been depleted from the supernatant (1-relative binding to pellet). All experiments were performed in triplicate. K_d_ values were calculated by plotting the relative binding vs the polysaccharide concentration in Prism (V7.0 GraphPad) and using the non-linear regression tool with the one-site binding hyperbola equation.

### 2.4 Antifungal assays

Antifungal assays were performed as described in (Bleackley et al., 2014). *S. cerevisiae* cells were prepared by diluting to an OD_600_ = 0.01 and *C. albicans* cells were diluted to OD_600_ = 0.0002 in ½ strength potato dextrose broth (1/2 PDB) (Becton Dickinson). Assays investigating the combined effect of chemical cell wall synthesis inhibitors with defensins were performed as described in (Bleackley et al., 2017). Synergy was assessed using the fractional inhibitory concentration (FIC) equation.

$$\mathrm{FIC}=\left(\frac{{\mathrm{MIC}}_{A}combination}{{\mathrm{MIC}}_{A}alone}\right)+\left(\frac{{\mathrm{MIC}}_{B}combination}{{\mathrm{MIC}}_{B}alone}\right)$$

Synergy is defined as an FIC value of less than 0.5. Assays investigating the activity of defensins in salt were set up using the same checkerboard method as the synergy assays but with NaCl in place of one of the antifungal molecules.

### 2.5 Activity of NaD1 against spheroplasts

Spheroplasts were prepared as outlined in (Guthrie and Fink, 2002) with modifications. Cells were prepared with and without the addition of zymolyase (Sigma) and washed twice with ½ PDB with 1 M sorbitol prior to any defensin treatment. Successful hydrolysis of the cell wall was assessed by pelleting an aliquot of spheroplasts, resuspending in MilliQ H_2_O and assessing cell lysis. For survival assays, intact cells or spheroplasts were diluted in ½ PDB with 1 M sorbitol to an OD_600_ = 1. NaD1 (10 µL) at 0, 25, 50 or 100 µM was added to 90 µL of prepared cells or spheroplasts and incubated at 30 ˚C for 30 min. Serial dilutions of each treatment were plated onto YPD and incubated for 24 h at 30 °C prior to imaging. Assessment of the effect of NaCl on the activity of NaD1 on spheroplasts was conducted as described above except for the addition of 100 mM NaCl to the ½ PDB 1 M sorbitol in the appropriate treatment. The cell death indicator, SYTOX green (ThermoFisher), was added to all treatments to a final concentration of 1 µM and FACS analysis was performed using a FACS Canto II (BD biosciences) using the FITC settings. Analysis was performed using Weasel V3.0 (Walter and Eliza Hall Institute of Medical Research).

### 2.6 Survival assays in the presence of exogenous 1,3-β-glucan

To assess the effect of exogenous 1,3-β-glucan on the activity of NaD1, a survival assay was employed essentially as described in (Bleackley et al., 2017). A two-fold dilution series of laminarin (Sigma) from 50 mg/mL was prepared in MilliQ H_2_O and 10 µL of each concentration plus a no laminarin control were transferred to the wells of a U-bottom 96 well microtitre plate (Greiner Bio-one). NaD1 (10 µL of 200 µM solution) was added to each laminarin preparation. Controls with no NaD1 or laminarin were also prepared. An overnight culture of *S. cerevisiae* BY4741 cells was diluted to OD_600_ = 0.1 in ½ PDB. The diluted yeast cell suspension was then added to each of the wells of the microtitre plate and incubated at 30 °C with shaking at 750 rpm in a Thermomixer (Eppendorff) for 1 h. A fivefold dilution series of each treatment was then spotted onto YPD agar (1% yeast extract, 2% peptone, 2% dextrose, 2% agar) and incubated at 30 °C prior to imaging.

### 2.7 NMR

The interaction between NaD1 and polysaccharides was characterized by NMR using ^15^N labelled NaD1 with laminarin (Sigma) as a source of β-glucan or chitohexaose (Megazyme) as a source of oligosaccharide from chitin. Spectra recorded at 600 MHz on a Bruker Avance I spectrometer included 2D ^15^N-Heteronuclear Single Quantum Coherence (HSQC), 3D ^15^N-HSQC-Nuclear Overhauser Effect spectroscopy (NOESY) and 3D ^15^N-HSQC-Total Correlation Spectroscopy (TOCSY). Since chemical shifts are indicative of local environment, titration experiments were conducted to determine changes that occurred upon the interaction of ^15^N-labelled NaD1 with laminarin or chitohexaose. Aliquots of a stock solution of laminarin (146 mg/mL H_2_O, 110 μL Na_3_PO_4_, 110 μL D_2_O) were sequentially titrated into an NMR tube containing the ^15^N-labelled NaD1 (400 μL ^15^N-NaD1 4 mg/mL 90% H_2_O/10% D_2_O v/v, 50 μL 0.5 M Na_3_PO_4_, pH 6.3). For the chitohexaose experiments, a stock solution of hexaacetyl chitohexaose (3.3 mg/mL H_2_O) was added similarly to a sample of ^15^N-labelled NaD1 (450 μL ^15^N-NaD1 1.1 mg/mL). Chemical shift differences of greater than 0.1 ppm (for ^15^N shifts) and 0.01 ppm (for proton shifts) were considered significant.

### 2.8 Molecular modelling

Relaxation of the NaD1 structure was performed by molecular dynamics (MD) simulation in GROMACS V 5.1.4 with the force field AMBER99SB-ILDN (Lindorff-Larsen et al., 2010). The structure of NaD1 (1MR4) (Lay et al., 2003b) was first solvated with 3600 TIP3P water molecules in a periodic octahedral box. The charge in the system was neutralised by the addition of 6Cl^-^ ions. The system was then subjected to energy minimisation using the steepest descent method as described by (Cerutti et al., 2009). The minimised system was then equilibrated over 150 ns in the NVT ensemble with 300 K followed by 300 ns of the NPT ensemble where 1 atm was used. Steps of 1 fs were using during the equilibration of the system. Equilibration steps and the MD production run were performed using the particle-mesh Ewald method for electrostatic interactions (Darden et al., 1993) and the LINCS algorithm (Hess et al., 1997) for constraint of hydrogen bonds. The production run was performed over 20 ns using a 2 fs time step. The simulation was run in triplicate using different random seeds. The MD production runs were performed using Raijin, a hybrid Fujitsu Primergy and Lenovo NeXtScale high-performance, distributed-memory cluster in the National Computational Infrastructure (NCI) at the Australian National University.

The average coordinates during the converged production runs were exported as a pdb structure file, which was the relaxed structure of NaD1.

### 2.9 Docking

The docking prediction was performed using Autodock VINA V1.1.2 (Trott and Olson, 2010) using the relaxed structure of NaD1 as the receptor molecule and chains of 3, 4, 5, or 6 units of chitin or 1-3 β-glucan as ligands. All bonds outside of the carbohydrate rings of the ligand were considered flexible. All of NaD1’s amino acid side chains, except for Cys, Pro, and Gly were considered flexible. The docking was run in a cubic box with 85 points per side that cover the surface of the peptide evenly. The docking run was performed using Raijin at NCI.

The number of polar contacts between NaD1 and carbohydrate amongst the top 4 poses for each ligand: receptor pair were quantified using PyMOL V1.8.6 (Schrödinger) with a cutoff of 3.5 Å.

## 3 Results

### 3.1 The plant defensin NaD1 binds to both chitin and β-glucan

**Fig. 1:**
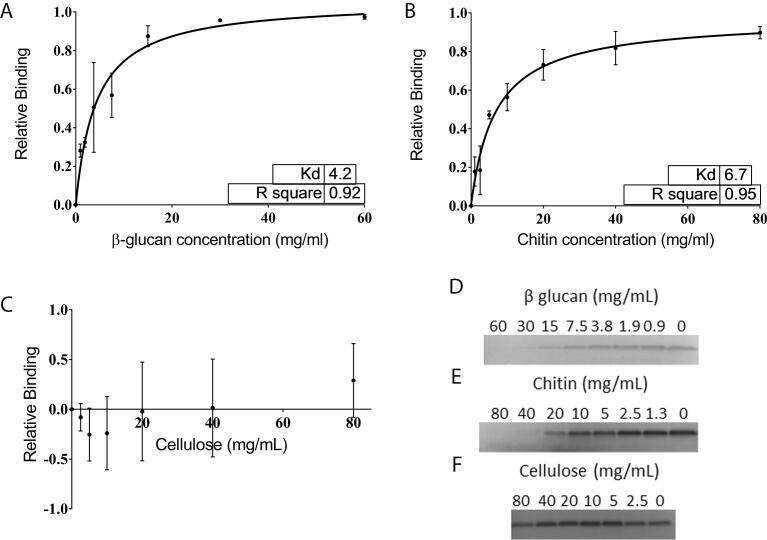
The interaction between NaD1 and β-glucan, chitin and cellulose. Binding of NaD1 to insoluble carbohydrates was assessed by solution depletion assays and SDS-PAGE analysis of supernatant. Binding isotherms for NaD1 binding to β-glucan (A), chitin (B) and cellulose (C) were generated from densitometry on three replicates of the SDS-PAGE analysis of supernatants from the defensin depletion assays. Gels were stained with RAPID stain. Representative gels are presented for β-glucan (D), chitin (E) and cellulose (F). The dissociation constants for each pair were calculated using a non-linear regression with a one site binding model. NaD1 binds chitin with a Kd of 6.7 ± 2.4 mg/mL and β glucan with a Kd of 4.2 ± 0.8 mg/mL which when calculated with respect to the molar concentration of each monosaccharide are 33.0 ± 11.8 mM and 25.9 ± 5.0 mM respectively. NaD1 did not bind to cellulose. Data is the average of 3 independent experiments. Error bars are standard deviation.

Binding of defensins to chitin, β-glucan and cellulose was determined using binding isotherm plots. The K_d_ and B_max_ were calculated using a one site binding model. Three independent replicates produced similar binding kinetics as indicated by an R^2^ value of greater than 0.9 for each experiment. The K_d_ s normalized for the concentration of the monosaccharide for chitin and β-glucan were 33.0 ± 11.8 mM and 25.9 ± 5 mM respectively. These values indicate that NaD1 has a similar affinity for β-glucan and chitin. NaD1 did not bind to the major plant cell wall polysaccharide, cellulose (Fig. 1).

### 3.2 The cell wall protects against the antifungal activity of NaD1

**Fig. 2:**
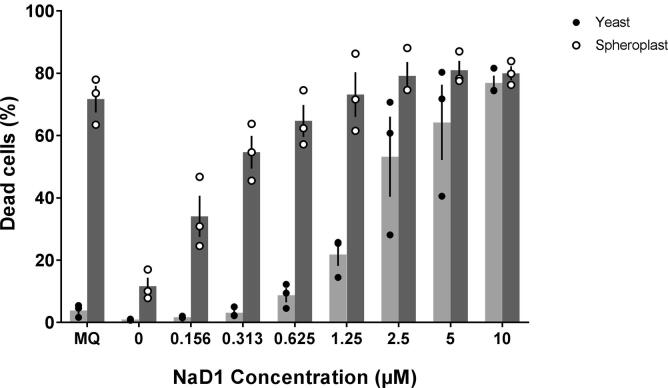
The cell wall protects yeast against the antifungal activity of NaD1. Intact yeast cells and spheroplasts were treated with a range of concentrations of NaD1 and cell death was monitored by flow cytometry using the cell viability stain SYTOX green. Cell death of spheroplasts (dark grey) occurred at much lower defensin concentrations than those required to kill intact yeast cells (light grey). Data is the average of three independent experiments, values for individual experiments are represented as filled (yeast) or empty (spheroplast) dots, error bars are standard error of the mean.

The requirement of cell wall polysaccharide for NaD1 activity on yeast was examined by comparing NaD1 activity on cells with intact cell walls to the activity on spheroplasts. Cell death occurred in over 60% the spheroplasts that had been treated with NaD1 at concentrations above 0.625 µM. In contrast, 60% death of cells with intact cell walls did not occur until the NaD1 concentration was raised to 5 µM (Fig. 2). Thus, the cell wall is not essential for the toxic effects of NaD1 on yeast. Indeed the cell wall protected the yeast cell against the antifungal activity of NaD1 because spheroplasts were killed by lower concentrations of NaD1 than cells with intact walls.

### 3.3 NaD1 is more active against fungal cells with decreased β-glucan but not less chitin in their cell walls

**Fig. 3:**
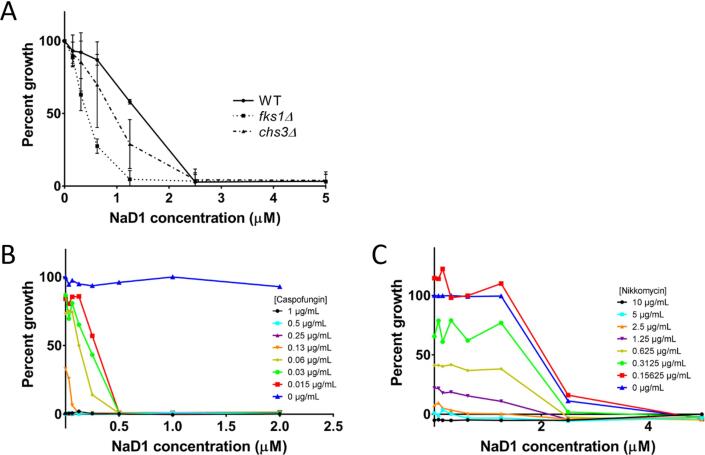
β-glucan and not chitin levels are responsible for the protective qualities of the fungal cell wall against plant defensins. The contributions of chitin and β-glucan towards the protective function of the fungal cell wall were assessed by modulating the level of each polysaccharide both chemically and genetically. (A) A *S. cerevisiae* strain with a deletion in the major 1,3-β-glucan synthase subunit *fks1*Δ was more sensitive to NaD1 than wild type or a strain lacking the major chitin synthase *chs3*Δ. Similarly, the 1,3-β-glucan synthase inhibitor caspofungin acted in synergy with NaD1 against *C. albicans* (B) while the chitin synthase inhibitor Nikkomycin Z did not (C).

The levels of either β-glucan or chitin in the yeast cell wall were lowered using both genetic and chemical means. The activity of NaD1 on *S. cerevisiae* strains with deletions in either *fks1*, which is proposed to be the major 1,3-β-glucan synthase, or *chs3*, the chitin synthase responsible for production of chitin at sites other than the bud neck, was assessed by comparing the growth of each strain to wild type over a range of concentrations of NaD1. The minimum inhibitory concentrations (MIC) is defined as the lowest concentration where no growth is observed. The MIC for the *fks1*Δ strain was 1.25 µM while the MICs for *chs3*Δ and the wild type were 2.5 µM indicating that *fks1*Δ but not *chs3*Δ was more sensitive to the defensin than wild type cells (Fig. 3A). The growth rate of *chs3*Δ was slightly lower than wild type cells at sub-inhibitory concentrations of NaD1 but this was not reflected in a change in MIC indicating that chitin levels in the cell wall do not dramatically affect susceptibility to NaD1.

To further evaluate the effect of decreased amounts of 1,3-β-glucan and chitin in the cell wall on NaD1 activity, the polysaccharide synthesis inhibitors caspofungin and nikkomycin Z were assessed for their impact on the activity of NaD1 against the human fungal pathogen *C. albicans*. Caspofungin and nikkomycin Z are inhibitors of 1,3-β-glucan synthesis and chitin synthesis respectively and treatment of fungi with these molecules leads to a decrease in the amount of β-glucan and chitin in the cell wall (Lee et al., 2018, Verwer et al., 2012). Potential synergy with these cell wall disruptors was assessed using standard checkerboard assays and determining the fractional inhibitory concentration (FIC) for each combination; with synergy defined as any combinations with an FIC less than 0.5. Concentration dependent growth inhibition was observed with the individual polysaccharide synthesis inhibitors as well as NaD1. The presence of caspofungin at concentrations as low as 0.015 µg/mL, which is 6% of the MIC (0.25 µg/mL), decreased the MIC of NaD1 dramatically as indicated by the FIC value of 0.3 (Fig. 3B). In contrast, nikkomycin Z had only a minimal effect on the antifungal activity of NaD1 even at concentrations close to the MIC (2.5 µg/mL) (Fig. 3C) as indicated by the FIC value of 0.92. This pattern was consistent with the activity of NaD1 against the polysaccharide synthesis mutants. In summary, decreasing 1,3-β-glucan levels in the yeast cell wall increases the activity of NaD1 while a decrease in chitin levels has minimal effect on NaD1 activity.

### 3.4 Exogenous 1,3-β-glucan protects against the activity of NaD1

**Fig. 4:**
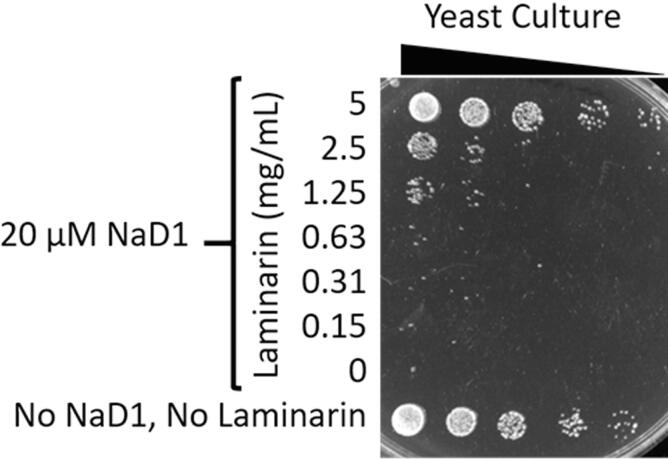
Exogenous 1,3-β-glucan protects yeast cells against the antifungal activity of NaD1. Yeast were treated with 20 µM NaD1 in the presence of a range of concentrations of laminarin, a soluble form of 1,3-β-glucan, for 1 h before a dilution series from each treatment was plated on YPD agar. In the absence of laminarin there are no viable cells remaining. At concentrations of 1.25 mg/mL and above laminarin had a protective effect against NaD1 as indicated by the presence of viable yeast cells.

*S. cerevisiae* cells were treated with 20 µM NaD1 in the presence of a range of concentrations of laminarin, a soluble form of 1,3-β-glucan with some 1,6 branch points and assayed for survival by plating on YPD agar. In the absence of laminarin, 20 µM NaD1 eliminated all viable yeast cells. Laminarin at concentrations above 1.25 mg/mL protected against the antifungal activity of NaD1 (Fig. 4). Thus exogenous 1,3-β-glucan can protect against the antifungal activity of NaD1.

### 3.5 Binding to fungal cell wall carbohydrates causes small shifts in the NaD1 peptide backbone

**Fig. 5:**
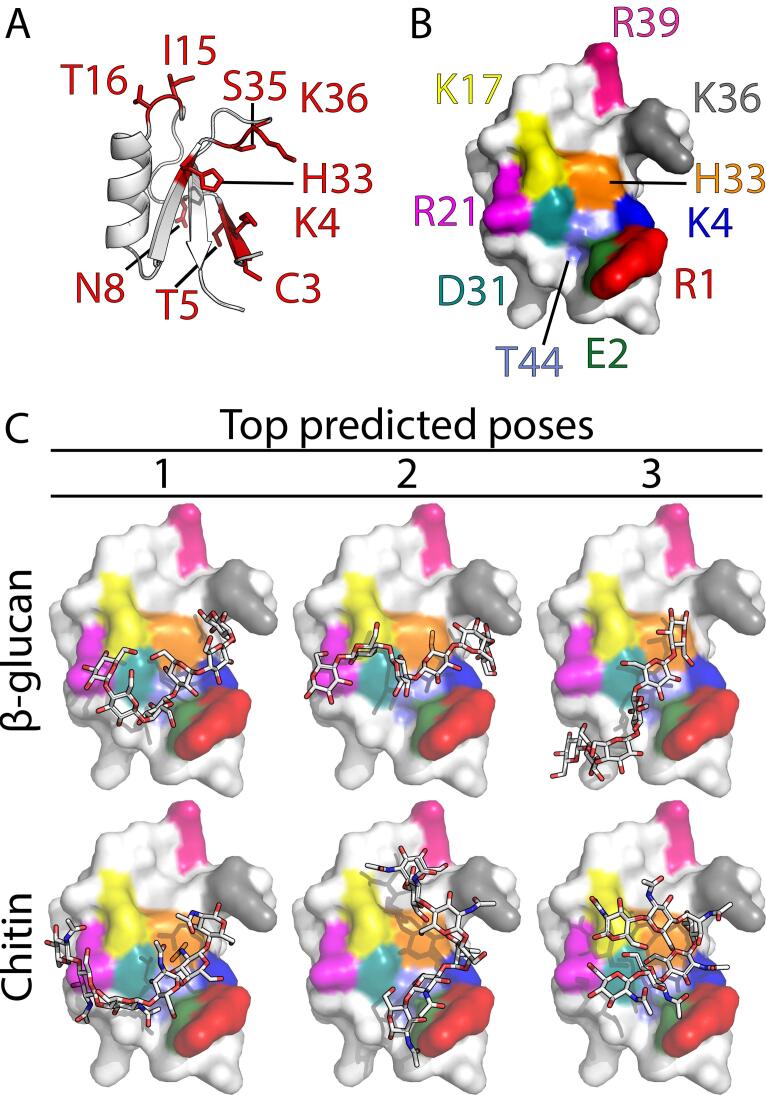
NMR Spectroscopy and molecular modelling identification of the interaction between NaD1 and 1,3-β-glucan or chitin. (A) NMR structure of NaD1 highlighting regions of secondary structure. Residues for which chemical shifts changes were observed when laminarin was added are shown in red. (B) The NMR structure of NaD1 (PDB 1mr4) was solvated and energy minimized and then used for docking studies with oligosaccharides of DP6. Residues found to be involved in carbohydrate binding are highlighted in colour. The top three scoring poses from each docking simulation with the DP 6 oligosaccharide are presented. Models of the docking of shorter oligosaccharides can be found in Fig. S2. (C) Top predicted poses of NaD1 with 1,3-β-glucan and chitin. (For interpretation of the references to colour in this figure legend, the reader is referred to the web version of this article.)

**Supplementary figure 1:**
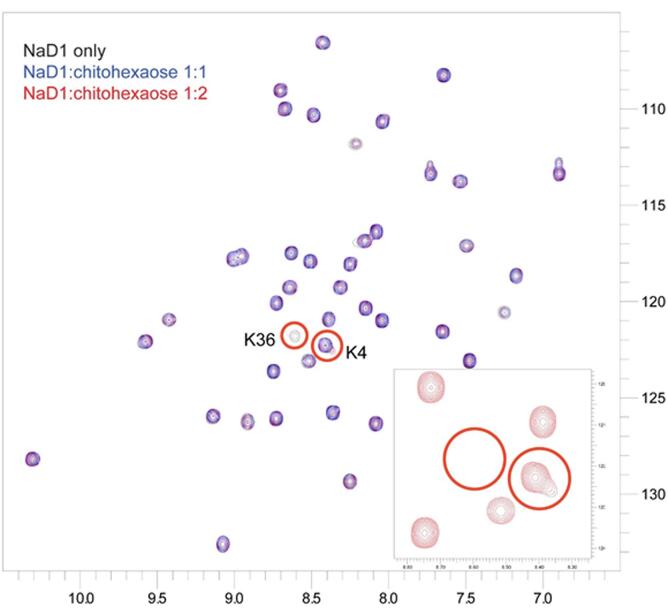


To identify which part of NaD1 is involved in the interaction with cell wall carbohydrates we used NMR spectroscopy to monitor the effects of addition of laminarin and chitohexaose to ^15^N-labelled NaD1. The titration of laminarin into the NaD1 sample affected a number of the backbone amide chemical shifts of NaD1. The effects were small, but consistent with an interaction involving the side chains of amino acid residues, since the NMR titration monitors primarily the backbone amides. The largest change seen for an ^15^N resonance was 0.199 ppm for I15 and for a proton resonance, the largest shift was 0.046 ppm for K36. These and other significant changes are listed in Table S1. The positions of the affected resonances are shown in Fig. 5A, which highlights the surface created by the triple stranded β-sheet as the binding site. When chitohexaose was added to NaD1, very little change was observed in resonance shifts even in the presence of up to 10-fold excess of ligand, but some selective broadening was observed. Fig. S1 illustrates the lack of changes in chemical shifts of all residues, but broadening of K36, whose signal disappeared completely, and of K4.

### 3.6 A molecular dynamics model of the NaD1 interaction with fungal cell wall carbohydrates

**Fig. 6:**
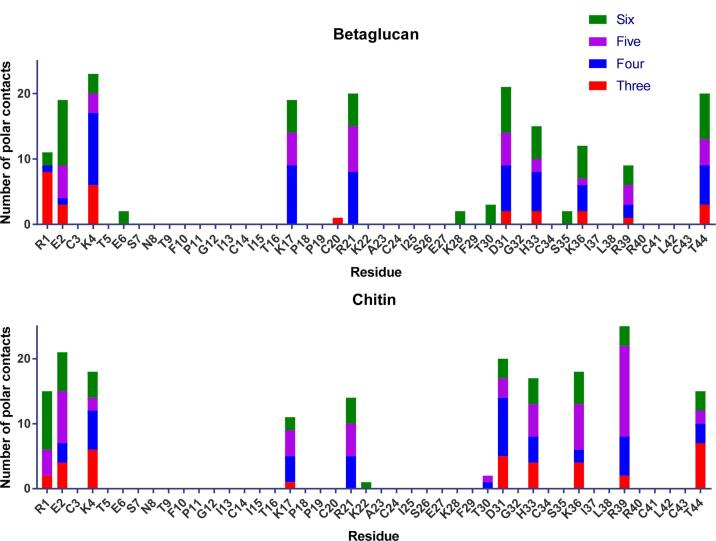
Density plot of polar contacts between ligand and receptor pairs. The number of polar contacts between the top four poses of NaD1 and the polysaccharides with DP of six (green), five (purple), four (blue) or three (red). The X axis indicates the residues of NaD1 that are involved in the interaction. (For interpretation of the references to colour in this figure legend, the reader is referred to the web version of this article.)

**Supplementary figure 2:**
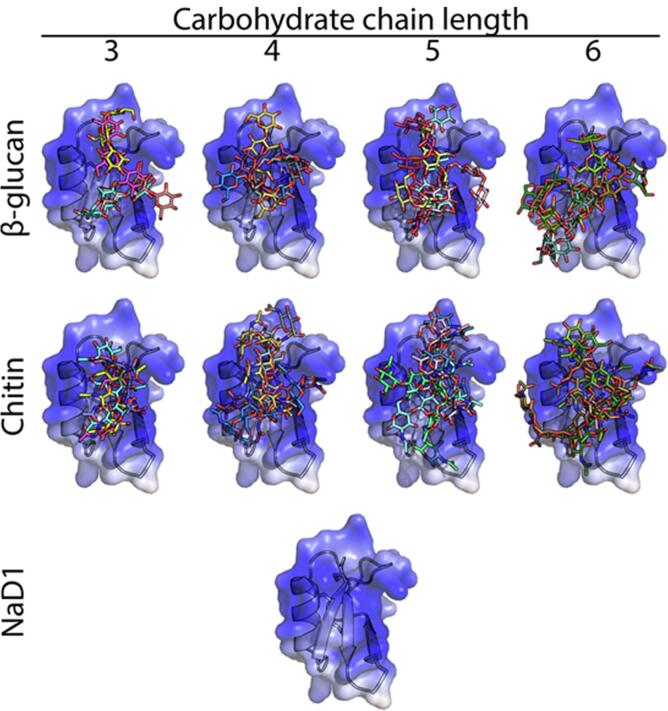


To generate a more detailed model of how the interaction between NaD1 and cell wall polysaccharides would occur the NMR structure of NaD1 (PDB ID 1mr4) was energy minimised, solvated and relaxed in molecular dynamics simulations (Fig. 5B). The relaxed structure obtained from this work was subsequently used for docking trials with chitin, and β-glucan oligosaccharides with a degree of polymerisation (DP) of 3, 4, 5 or 6. Each of the ligand: receptor docking pairs were run multiple times using different random seeds to ensure the results observed were not dependent on the initial conditions. Models of NaD1 and either carbohydrate implicated the same region of NaD1 as responsible for binding. Importantly this region was fully consistent with the NMR data. The modelling with trisaccharide chains also indicated overlap between the position of monosaccharide residues at the reducing and non-reducing ends of the polysaccharide indicating that it is likely that a long chain oligosaccharide would adopt a similar conformation (Fig. S2). Indeed, the models obtained with larger oligosaccharides were consistent with the trisaccharides and with each other (Fig. 5C, S2). However, whether the models of the interaction with oligosaccharides truly represent the interaction with an actual polysaccharide chain as would be found in the cell wall is speculative. The ligand poses with the four best binding energies were evaluated in PyMOL where polar contacts between the monosaccharide residues and the defensin side chains were counted. These polar contacts were consistent between both chitin and β-glucan molecules (Fig. 6) and revealed that the basic residues R1, K4, K17, R21, K36 and H33 are responsible for carbohydrate binding with R39 also prominent in the binding of chitin (Fig. 6). The acidic residues E2 and D31 and terminal residues R1 and T44 also had multiple polar contacts with the ligands.

### 3.7 Fungal cell wall polysaccharide binding by other plant defensins

To determine whether the interaction with fungal cell wall polysaccharides was unique to NaD1 or was a common feature of plant defensins the chitin and yeast β-glucan binding assays were performed using a second defensin from *N. alata* (NaD2), a defensin from dandelion (TsD10), a defensin from *Dahlia merckii* (DmAMP1) and an artificial chimeric defensin (HXP4) (Table 1). HXP4 bound to β-glucan with a similar K_d_ to NaD1 with NaD2 having a higher K_d_ and TsD10 higher again. No binding of DmAMP1 to β-glucan was detected. Both NaD2 and HXP4 bound to chitin with a higher affinity than NaD1. Neither TsD10 nor DmAMP1 bound to chitin. The R^2^ values for all the binding isotherms where binding was detected were above 0.85 indicating a good fit for each binding isotherm.

**Table 1:** Binding of defensins to fungal cell wall polysaccharides. Binding was assessed using binding isotherms as presented in Fig. 1 for NaD1. Data presented is calculated from the average of three independent experiments. NB indicates combinations where no binding was observed. K_d_ values are calculated based on the concentration of the monosaccharide units as the polysaccharides used were heterogeneous and are presented ± SEM from three independent replicates.

Polysaccharide		NaD1	HXP4	NaD2	TsD10	DmAMP1
Β-glucan	K_d_ (mM)	25.9 ± 5.0	30.2 ± 12.0	58.6 ± 11.7	202.5 ± 25.9	NB
	R-squared	0.92	0.94	0.88	0.87	NB
Chitin	K_d_ (mM)	33.0 ± 11.8	6.9 ± 3.0	7.9 ± 2.0	NB	NB
	R-squared	0.95	0.96	0.97	NB	NB

### 3.8 Effect of decreasing cell wall 1,3-β-glucan and chitin on the activity of other plant defensins

The effect of a decrease in the levels of cell wall 1,3-β-glucan and chitin on the activity of the other 4 plant defensins listed above was assessed using the deletion strains and synergy assays with caspofungin and nikkomycin Z as described for NaD1. All the defensins had a 2-fold lower MIC against *fks1*Δ compared to wild type and only TsD10 was more active against *chs3*Δ (Table 2). Similarly, caspofungin enhanced the activity of all the defensins much better than nikkomycin Z. All the defensins except DmAMP1 had an FIC value of less than 0.5 in combination with caspofungin indicating a strong synergistic interaction. None of the defensins had an FIC of less than 0.5 when used in combination with nikkomycin Z (Table 3).

**Table 2:** MIC in µM of defensins against cell wall polysaccharide synthesis mutants. MIC values were consistent across three independent experiments where a twofold dilution series of defensin was tested in a fungal growth assay. The MIC was defined as the lowest concentration where there was no visible growth.

Strain	NaD1 (µM)	HXP4 (µM)	NaD2 (µM)	TsD10 (µM)	DmAMP1 (µM)
BY4741	2.5	2.5	5	20	0.31
*fks1*Δ	1.25	1.25	1.25	10	0.16
*chs3*Δ	2.5	2.5	5	10	0.31

**Table 3:** Synergy between polysaccharide synthase inhibitors and plant defensins. Checkerboard assays against *S. cerevisiae* were used to assess synergy between defensins and the polysaccharide synthase inhibitors caspofungin and nikkomycin. FIC values for each defensin-inhibitor combination are presented as the average of three independent experiments plus or minus the standard deviation. Synergy is characterized as an FIC value of <0.5.

	NaD1	HXP4	NaD2	TsD10	DmAMP1
Caspofungin	0.3 ± 0.06	0.3 ± 0.04	0.2 ± 0.05	0.3 ± 0.04	0.6 ± 0.06
Nikkomycin	0.55 ± 0.02	1 ± 0	1 ± 0	0.57 ± 0.08	0.64 ± 0.16

### 3.9 The effect of salt on the antifungal activity of NaD1 is dependent on the cell wall

**Fig. 7:**
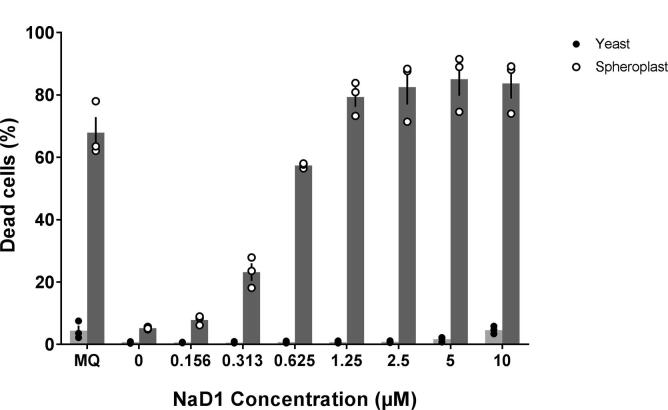
Loss of NaD1 activity in the presence of 100 mM NaCl is dependent on the cell wall. Cell death following treatment of spheroplasts and intact cells was measured using flow cytometry after staining with the cell viability marker SYTOX green. NaD1 was much more active on spheroplasts (dark grey) than yeast cells with intact cell walls (light grey) in the presence of 100 mM NaCl. Data is the average of three independent experiments, values for individual experiments are represented as filled (yeast) or empty (spheroplast) dots, error bars are standard error of the mean.

NaD1 is active against both fungal and human lymphoma cells. However, antifungal activity is blocked in the presence of NaCl whereas the activity on human lymphoma cells is not affected by high salt solutions such as RPMI medium or PBS (Poon et al., 2014). This led to the hypothesis that it is the cell wall that blocks the antifungal activity of NaD1 when salt concentrations are above 100 mM. Spheroplasts and yeast cells with intact cell walls were thus suspended in 1 M sorbitol with 100 mM NaCl before exposure to various concentrations of NaD1 (Fig. 7). Cell death was monitored by the inclusion of the cell death stain SYTOX green. In 100 mM NaCl there was significant cell death of spheroplasts at NaD1 concentrations of 0.313 µM and higher, whereas cells with intact cell walls were unaffected up to 10 µM NaD1 (Fig. 7). These results support the hypothesis that the cell wall has a key role in repression of the antifungal activity of NaD1 in the presence of salt.

### 3.10 Defensins that do not bind cell wall polysaccharides are salt tolerant

**Fig. 8:**
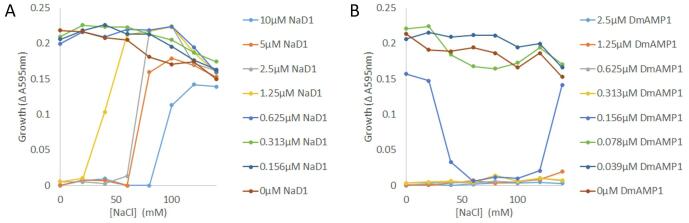
Unlike NaD1, DmAMP1 does not lose activity at higher salt concentrations. *S. cerevisiae* was grown in ½ PDB with increasing concentrations of salt and plant defensins in a checkerboard pattern. Growth at each defensin concentration was plotted as a function of salt concentration. (A) The activity of NaD1 against *S. cerevisiae* was inhibited by increasing concentrations of salt as indicated by the increase in absorbance at 595 nm in the 1.25, 2.5, 5 and 10 µM traces as the NaCl concentration approached 100 mM. (B) DmAMP1 activity was not inhibited by the NaCl as indicated by the lack of increase in absorbance at 595 nm in the 0.313, 0.625, 1.25 and 2.5 µM traces. Intermediate concentrations of NaCl improved the growth inhibition by 0.156 µM DmAMP1. Data is representative of three independent replicates.

As the cell wall mediates the loss of antifungal activity of NaD1 at elevated salt concentrations we hypothesized that defensins such as DmAMP1 that do not bind to cell wall polysaccharides would be salt tolerant. This was tested in growth inhibition assays with *S. cerevisiae* using NaD1 or DmAMP1 in media with varying concentrations of NaCl (Fig. 8). In the absence of added NaCl, NaD1 completely inhibited *S. cerevisiae* growth at concentrations of 1.25 µM and above. As the NaCl concentration was increased, NaD1 became a progressively poorer inhibitor. This inhibition of NaD1 by salt was dependent on the concentration of NaD1, that is, the higher the concentration of NaD1 the more salt required to block the antifungal activity. In comparison DmAMP1 also inhibited growth of *S. cerevisiae* with no growth at 0.313 µM DmAMP1 in the absence of salt. However, there was no loss of growth inhibitory activity when NaCl concentrations were increased to levels above those required to block NaD1 activity. In fact, 0.156 µM DmAMP1 was more active against yeast cells at intermediate concentrations of NaCl. Thus, DmAMP1, the defensin that did not interact with fungal cell wall polysaccharides is more salt tolerant than NaD1.

## 4 Discussion

The cell wall is the first contact point between antifungal molecules and fungal cells. In this study we show that the plant defensin NaD1 interacts with β-glucan and chitin, the two main polysaccharides in the fungal cell wall. Merely identifying this interaction did not resolve whether the cell wall functions to protect the fungus against the activity of NaD1, or if it is a receptor that effectively concentrates the defensin on the surface of fungal cells and thus facilitates the antifungal activity. Removal of the cell wall by hydrolysis with zymolyase increased the susceptibility of *S. cerevisiae* cells to the cytotoxic effects of NaD1. At first this result appeared to contradict previous work with *F. oxysporum* where treatment of the mycelium with β-glucanase or proteinase K prevented NaD1 from killing hyphae (van der Weerden et al., 2010). However, these cells had retained enough cell wall structure to maintain their filamentous morphology and it is likely the treatment induced stress responses that triggered reinforcement of the cell wall (Hayes et al., 2014). These experiments were performed using a concentration of NaD1 close to the MIC. Thus a relatively small decrease in susceptibility could have resulted in what appeared to be a lack of activity on cells treated with β-glucanase or proteinase K. One of the components of the antifungal mechanism of NaD1 is permeabilization of the fungal plasma membrane (van der Weerden et al., 2010) and one of the major functions of the cell wall is to provide mechanical support to the membrane. Thus, it was not surprising that removal of the cell wall described here increased the susceptibility of yeast cells to NaD1. Furthermore, the interaction between NaD1 and cell wall polysaccharides is likely to augment the protective effect of the cell wall by impeding the access of the defensin to the membrane and thus slowing uptake into the cell where it mediates its toxic effects. The binding affinities of the defensins assessed here (K_d_ in the range of 10^−3^ M) for fungal cell wall polysaccharides is lower than those reported for lectins and their respective carbohydrate-binding domains (K_d_ range from 10^−4^–10^−8^ M) (Duverger et al., 2003). The affinity of defensins for fungal cell wall polysaccharides is also lower than those reported for proteins such as CERK1 and other LYSM PRR proteins from *A. thaliana* that bind to oligosaccharides derived from the fungal cell wall to sense the pathogen and initiate a host immune response (K_d_ range from 10^−4^ to 10^−5^) (Cao et al., 2014, Mélida et al., 2018). The lower affinity of defensins reflects that binding to the fungal cell wall polysaccharides merely retards defensin activity as the proteins dissociate and subsequently bind to their membrane targets whereas with lectins and other receptor proteins, carbohydrate binding is the endpoint. The transient interaction between defensins and the fungal cell wall is also supported by the rapid cell surface localization of fluorescently labelled NaD1 in confocal microscopy experiments with *C. albicans* (Hayes et al., 2013) and *F. oxysporum* (van der Weerden et al., 2008) that occurs before translocation of the defensin to the cytoplasm and fungal cell death.

**Fig. 9:**
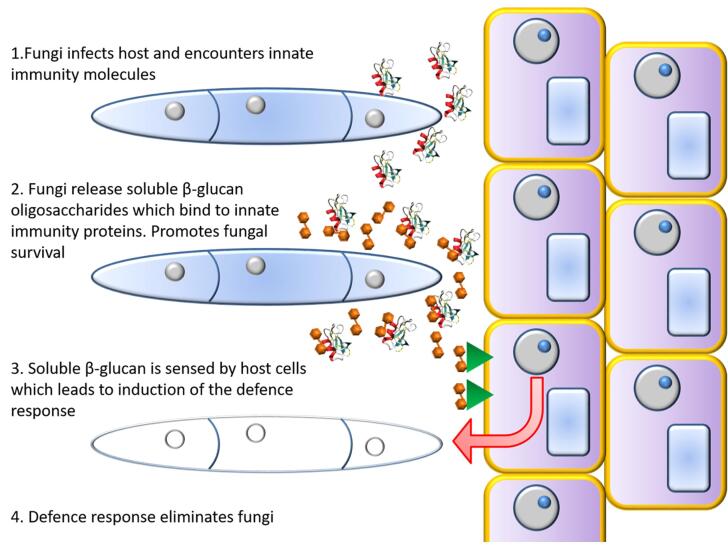
The proposed role of shed soluble β-glucan in the interaction between fungi and the host immune response. When fungi initially invade a host, they encounter a suite of innate immunity molecules which are toxic to the fungi. In response the fungi release soluble β-glucan which in turn binds to the innate immune molecules rendering them inactive. The host has evolved to exploit the release of soluble β-glucan as a signal associated with fungal invasion and mounts a further defence response which may eliminate the fungus.

1,3-β-Glucans have been detected in the serum of patients with a range of fungal infections in humans (Miyazaki et al., 1995) and in the apoplastic fluid of plants undergoing fungal infections (Wawra et al., 2016). In both mammals and plants, oligosaccharides released from fungal β-glucans act as pathogen associated molecular patterns (PAMPs) that activate defence responses via mechanisms that include dectin-1 in humans (Brown and Gordon, 2005) and FGB1 (Wawra et al., 2016) and others in plants (Fesel and Zuccaro, 2016). The protective effect of exogenous β-glucan against the antifungal effects of NaD1 led us to consider that the release of β-glucan by fungi may protect fungal pathogens from host innate immunity molecules during the initial stages of an infection and that the host responds by detection of the shed oligosaccharides as PAMPs and induction of further defence responses (Fig. 9). The data presented here is limited to plant defensins but human innate immunity proteins with antifungal activity such as LL37 and Histatin 5 have also been reported to bind to β-glucans (Jang et al., 2010, Tsai et al., 2011) and upregulate β-1,3-exoglucanase activity in *C. albicans* (Chang et al., 2012).

Modelling of the interaction between NaD1 and oligosaccharides of chitin and 1,3-β-glucan using molecular dynamics and docking predictions revealed that the same residues (R1, E2, K4, K17, R21, D31, H33, K36 and T44) are predicted to facilitate the interaction with longer oligosaccharides from both chitin and β-glucan and possibly with longer chains of the glycans. The involvement of many of these contacts was experimentally confirmed by NMR, which highlighted changes to the backbone amide resonances when laminarin or chitohexaose was added to samples of NaD1. The broadening and complex line shape seen in the case of chitin and the effects on chemical shifts observed for B-glucan suggest there might be differences in the exchange rates under these conditions. Both the observed line shape changes and chemical shift perturbations of the backbone amide resonances are small, but this is not unexpected considering that the modelled interactions involve polar contacts between the oligosaccharides and the exposed side chains of the peptide, and thus are not likely to cause significant backbone structural rearrangements.

There is significant overlap between the amino acid side chains that are predicted to make polar contacts between NaD1 and 1,3-β-glucan and the residues that mediate the interaction between NaD1 and phosphatidylinositol 4,5 bisphosphate (PI(4,5)P_2_), the main lipid binding partner of NaD1 (Poon et al., 2014). Three of the six key residues involved in PI(4,5)P_2_ binding, namely K4, H33 and K36, are also involved in the interaction with cell wall polysaccharides. Additional residues (R1, E2, K17, R21, D31 and T44) aside from those involved in lipid binding were also involved in the modelled interaction with the oligosaccharides. Except for T44 all the amino acids involved in the interaction carry a charge indicating that the interactions between NaD1 and the fungal cell wall are largely electrostatic. This interaction is different from the interaction between the mammalian immunity β-glucan receptor Dectin-1 where the protein-polysaccharide interaction is mediated through hydrophobic contacts (Brown et al., 2007). However, electrostatic interactions between β-glucan and a protein do contribute to the recognition of fungal β-glucans by the insect β-glucan receptor protein (Kanagawa et al., 2011). The role of electrostatics in the interaction between NaD1 and fungal cell wall polysaccharides is further supported by examination of the amino acid residues that occupy the equivalent positions in the non-polysaccharide binding defensin DmAMP1 (Fig. S3A). The only residues with conserved charge in both NaD1 and DmAMP1 are R39/R38 and potentially K36/H36 depending on the pH. All the other residues identified as polar contacts between NaD1 and 1,3-β-glucan or chitin have the opposite charge or no charge in DmAMP1. When the solvent accessible surface charge of a model of DmAMP1 is compared to that of NaD1 there are more negatively charged and neutral surfaces (Fig. S3B).

The contribution of the major cell wall polysaccharides towards the protective role of the cell wall against NaD1 was examined by decreasing the levels of either chitin and 1,3-β-glucan by genetic or chemical means. Decreasing 1,3-β-glucan by deletion of the gene encoding the major synthase *fks1* or by inhibition of Fks1p with caspofungin (Lee et al., 2018) increased the activity of NaD1 significantly while decreasing chitin levels had no effect. Caspofungin had a more of an effect on the activity of NaD1 than deletion of *fks1* due to the presence of additional 1,3-β-glucan synthases such as *FKS2* in the yeast genome (Mazur et al., 1995) that are also inhibited by caspofungin but are still active in the *fks1* deletion mutant. A similar pattern was observed for most of the other plant defensins assayed in this study. This is consistent with previous reports on the synergistic activity of the plant defensins HsAFP1, RsAFP1 and RsAFP2 with caspofungin (Vriens et al., 2016, Vriens et al., 2015). However, the increased activity of defensins on cells with decreased 1,3-β-glucan was not entirely dependent on interaction with β-glucan because DmAMP1, which does not interact with β-glucan, was also more active on *fks1*Δ compared to wild type and additionally was more active in the presence of caspofungin. However, the magnitude of the improvement of DmAMP1 activity in the presence of caspofungin was less than for the other defensins. These results indicate that the 1,3-β-glucan in the cell wall protects against defensin activity by both binding to defensins to prevent them from accessing the membrane and providing mechanical support to the membrane to protect against membrane damage.

One of the major hurdles in the clinical development of antimicrobial peptides, including AFPs, for use in the treatment of disease in humans is their lack of activity at physiological salt concentrations (Mohanram and Bhattacharjya, 2016, Yu et al., 2011). There are reports of salt tolerant AMPs, mostly from marine organisms (Fedders et al., 2008, Lee et al., 1997), but they are uncommon. The apoplastic fluid of most plants contains a lower concentration of monovalent cations than human serum with [K^+^] in plants ranging from ∼4–20 mM (Gabriel and Kesselmeier, 1999, Lohaus et al., 2001) and [Na^+^] in human serum ranging from 136 to 145 mM (Adrogué and Madias, 2000a, Adrogué and Madias, 2000b) (Na^+^ is not essential for plant growth but K^+^ is, making K^+^ the major cation in plant fluids (Maathuis, 2014)). The lower cation concentration in plants means that their antimicrobial defence molecules have had no selective pressure to retain activity at salt concentrations equivalent to those in human extracellular fluids. NaD1 is one of the many peptides that lose antifungal activity when salt concentrations are elevated. However, NaD1 is active against tumour cells and binds liposomes and lipid bilayers at salt concentrations at or above 100 mM (Payne et al., 2016, Poon et al., 2014). This led to the hypothesis that the cell wall was responsible for the inactivation of cationic AFPs such as NaD1 when NaCl concentrations were raised to 100 mM or above. Hydrolysis of the cell wall by zymolyase treatment restored the activity of NaD1 in 100 mM NaCl confirming that the effect of salt on defensin activity is mediated by the fungal cell wall. Whether it is a physical change in the packing of cell wall polymers at higher salt concentrations as observed when *C. albicans* are shifted to high salt conditions (Ene et al., 2015) that decreases the porosity of the wall or simply that the increase in ionic strength interferes with the ionic interaction between the defensin and outer surface of the cell wall remains to be elucidated. That NaD1 retains activity in the presence of 1 M sorbitol indicates that the loss of activity in salt is likely the result of electrostatic interactions as sorbitol has similar effect on cell wall ultrastructure as NaCl (Ene et al., 2015). Electrostatic interactions contribute to the antifungal activity of another plant antifungal protein Osmotin. Yeast strains with deletions in genes encoding proteins that add mannosylphosphate residues to cell wall mannoproteins, leading to a decreased negative charge on the cell wall, were resistant to Osmotin (Ibeas et al., 2000). The interaction between yeast phosphomannoproteins and NaD1 has not been examined. However, we have discovered that deletion of the major regulator of polyamine uptake in the yeast strain *agp2*Δ leads to a marked decrease in the negative charge on the cell surface that results in NaD1 resistance (Bleackley et al., 2014).

DmAMP1, the only defensin in our set that did not interact with either β-glucan or chitin is also a salt tolerant AFP. DmAMP1 acts by binding to mannosyl-diinositol-phosphorylceramide (M(IP)_2_C) in the cell wall and/or membrane (Thevissen et al., 2000). This interaction may not be affected by increased ionic strength. Alternatively, the lack of interaction between DmAMP1 and the major cell wall polysaccharides may allow the defensin to pass through the cell wall unhindered to access M(IP)_2_C in the plasma membrane.

Plant defensins are a large family of antifungal peptides that have potential for disease control in both agriculture and medicine. To realise their full potential, we must understand how they exert their activities on target fungal cells. Here the interactions between a set of defensins and fungal cell wall polysaccharides have been characterized. A strong synergistic activity between these defensins and caspofungin may have implications in the design of future antifungal treatment strategies. Most importantly we have identified why many cationic antifungal peptides lose activity at elevated salt concentrations. This discovery will inform the search for additional salt tolerant AFPs from naturally occurring sources and the design of synthetic AFPs with improved properties for commercial development.

## Declaration of Competing Interest

The authors declare that they have no known competing financial interests or personal relationships that could have appeared to influence the work reported in this paper.

## Figures and Tables

**Supplementary figure 3:**