# Supplementary material for: The interaction with fungal cell wall polysaccharides determines the salt tolerance of antifungal plant defensins
Source: Cell Surf. 2019 May 22;5:100026. doi: 10.1016/j.tcsw.2019.100026 (PMC7389181; doi:10.1016/j.tcsw.2019.100026)
Supplement: Supplementary data 1 [file mmc1.docx]

**Supplementary Information**

**Table S1. Chemical shifts of atoms of ^15^N-labelled NaD1 in the presence of laminarin as determined by NMR.** Chemical shift differences of greater than 0.1 ppm (for ^15^N shifts) and 0.01 ppm (for ^1^H shifts) were considered significant.

| Residue | Atom | 0 μL^a^ | 4 μL | 10 μL | 20 μL | 40 μL | 80 μL | Δδ (ppm) |
| --- | --- | --- | --- | --- | --- | --- | --- | --- |
| C3 | N | 125.40 | 125.40 | 125.37 | 125.33 | 125.29 | 125.21 | 0.19 |
| K4 | N | 122.28 | 122.28 | 122.28 | 122.24 | 122.20 | 122.10 | 0.18 |
| T5 | N | 120.07 | 120.06 | 120.06 | 120.04 | 119.96 | 119.89 | 0.18 |
| N8 | ND2 | 113.41 | 113.41 | 113.41 | 113.39 | 113.34 | 113.31 | 0.10 |
| N8 | HN | 10.221 | 10.216 | 10.216 | 10.210 | 10.210 | 10.198 | 0.023 |
| I15 | N | 126.46 | 126.46 | 126.49 | 126.50 | 126.57 | 126.66 | 0.20 |
| T16 | N | 111.68 | 111.67 | 111.67 | 111.64 | 111.59 | 111.48 | 0.20 |
| T16 | HN | 8.124 | 8.120 | 8.120 | 8.118 | 8.114 | 8.100 | 0.024 |
| H33 | N | 116.70 | 116.67 | 116.67 | 116.61 | 116.58 | 116.51 | 0.19 |
| S35 | N | 121.19 | 121.16 | 121.16 | 121.16 | 121.14 | 121.06 | 0.13 |
| K36 | N | 121.68 | 121.68 | 121.68 | 121.64 | 121.63 | 121.58 | 0.10 |
| K36 | HN | 8.575 | 8.569 | 8.567 | 8.566 | 8.554 | 8.529 | 0.046 |

^a^Added amount of laminarin stock solution

**Table S2**

**Binding energies for top ten poses of NaD1 with respective oligosaccaride in kcal/mol**

|  | **Chain length** | | | |
| --- | --- | --- | --- | --- |
| **1,3-β-glucan** | **3** | **4** | **5** | **6** |
|  | -7.3 | -7.1 | -5.4 | -5.8 |
|  | -7.3 | -6.8 | -5.3 | -5.6 |
|  | -7 | -6.3 | -5.3 | -5.5 |
|  | -7 | -6.3 | -5.2 | -5.3 |
|  | -7 | -6.2 | -5.2 | -5.3 |
|  | -6.9 | -6.2 | -5.2 | -5.1 |
|  | -6.9 | -6.1 | -5.2 | -5 |
|  | -6.9 | -6 | -5.1 | -5 |
|  | -6.9 | -6 | -5.1 | -5 |
|  | -6.8 | -5.8 | -5.1 | -5 |
|  |  |  |  |  |
| **Chitin** | **3** | **4** | **5** | **6** |
|  | -6.1 | -6.3 | -6.5 | -6.5 |
|  | -5.7 | -6.2 | -6.3 | -6 |
|  | -5.6 | -5.9 | -6.2 | -5.9 |
|  | -5.4 | -5.8 | -6.2 | -5.9 |
|  | -5.4 | -5.7 | -6.2 | -5.9 |
|  | -5.4 | -5.6 | -6.1 | -5.6 |
|  | -5.3 | -5.5 | -6.1 | -5.5 |
|  | -5.3 | -5.4 | -6 | -5.4 |
|  | -5.3 | -5.3 | -5.9 | -5.3 |
|  | -5.2 | -5.3 | -5.9 | -5.3 |


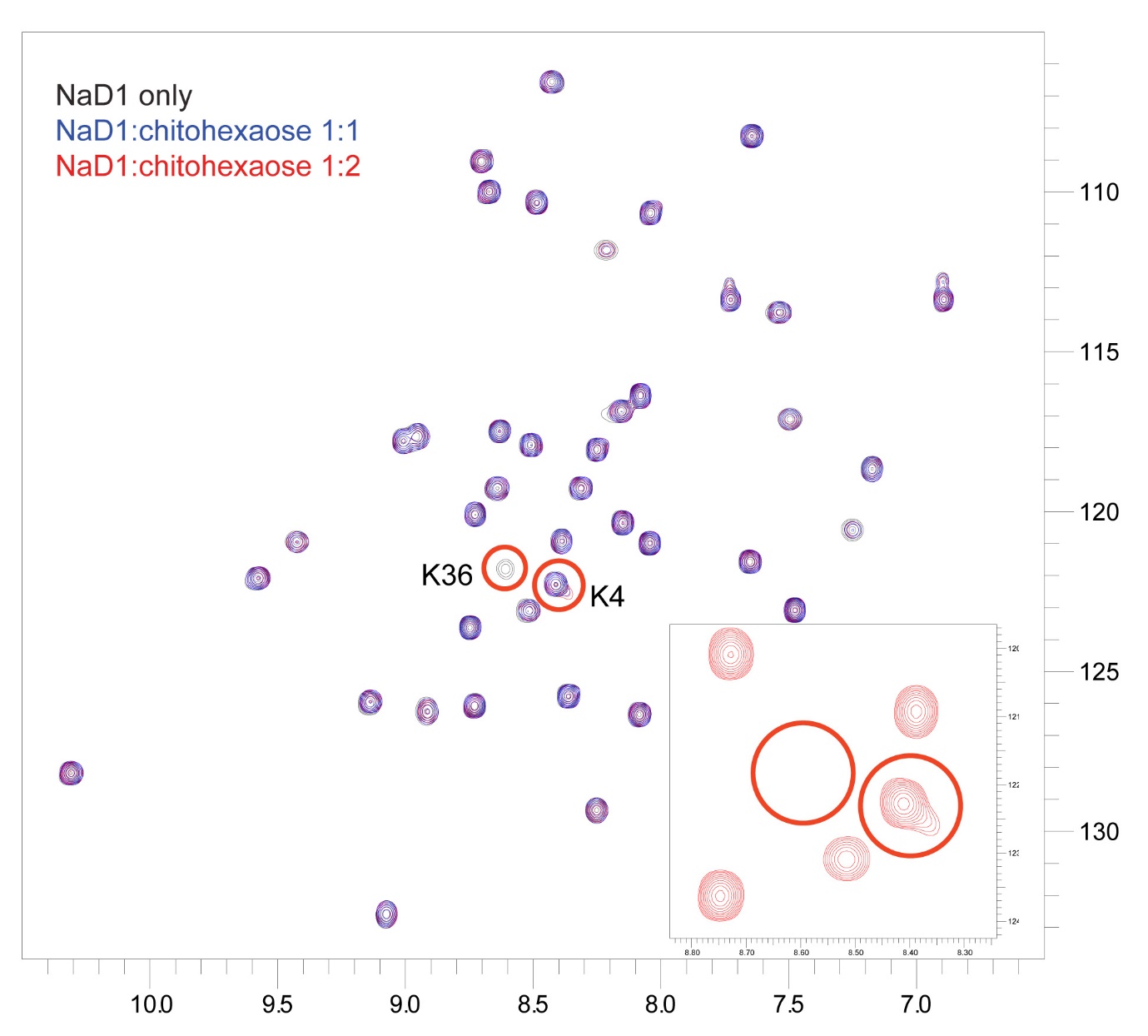


**Figure S1. Chitohexaose induces minimal structural change in NaD1.** ^1^H-^15^N HSQC spectra of NaD1 (black) with one-fold (blue) and two-fold (red) addition of chitohexaose. Spectra were acquired at 600 MHz, 303K. Affected residues are circled, labelled and shown in the expanded inset spectrum.


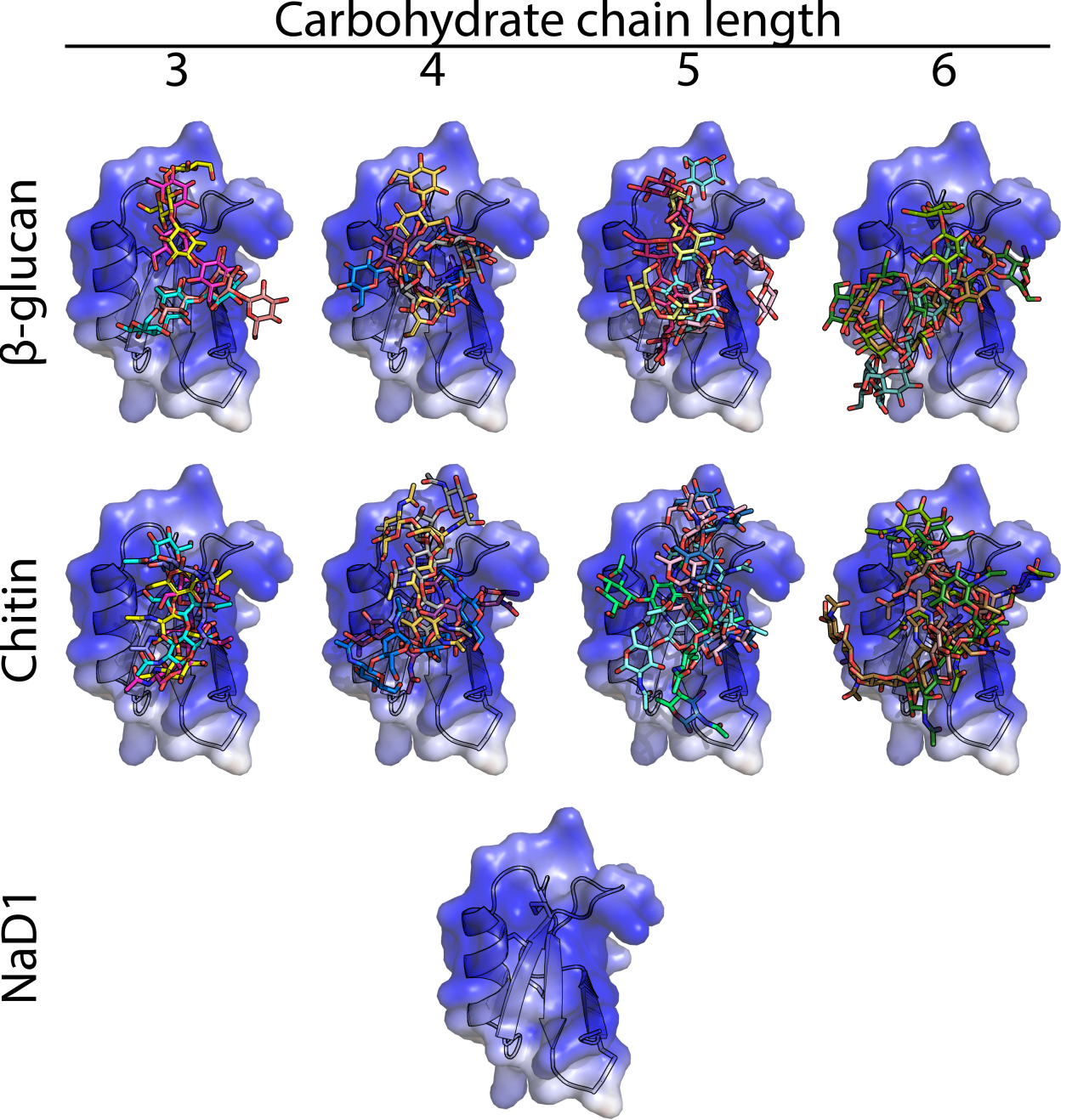


**Figure S2.** **Predicted docking of 1,3-β-glucan and chitin on NaD1 using oligo saccharides of varying lengths.** The predicted docking poses with the highest binding energies are illustrated for 1,3-β-glucan (top row) and chitin (bottom row) on the scaffold of the receptor defensin NaD1. The surface of NaD1 is displayed coloured according to the electric potential on the solvent. Only the top four poses are displayed for each ligand:receptor pair. The binding energies for the top ten poses of each oligosaccharide chain with NaD1 are listed in Table S2.

**
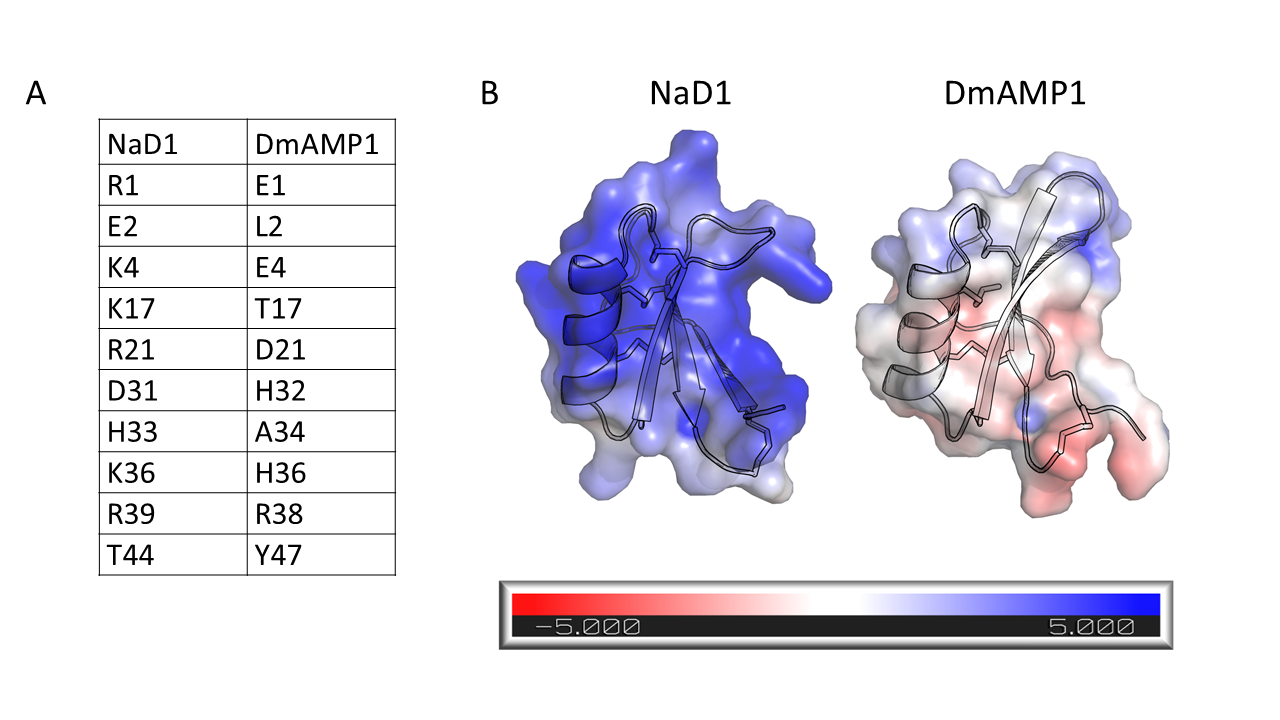
**

**Figure S3. Comparison of polysaccharide binding amino acids in NaD1 with amino acids in the equivalent positions in the non-polysaccharide binding defensin DmAMP1.** (A) Table of residues involved in polysaccharide binding in NaD1 as identified by NMR spectroscopy and molecular modeling and equivalent residues in DmAMP1. (B) Solvent accessible surface charge of NaD1 and DmAMP1. The DmAMP1 structure was made by homology modeling based on the NaD1 structure and refined using MD. The colors on the structure correspond to the charge as denoted on the bar at the bottom of the panel.
